# Microsurgical treatment of sacral spinal dural arteriovenous fistula

**DOI:** 10.3171/2025.7.FOCVID25112

**Published:** 2025-10-01

**Authors:** Hana Hallak, Ananth K. Vellimana

**Affiliations:** Department of Neurological Surgery, Washington University School of Medicine, St. Louis, Missouri

**Keywords:** spinal dural arteriovenous fistula, spinal dAVF, sacral dural arteriovenous fistula, sacral dAVF, lateral sacral artery dAVF

## Abstract

Sacral spinal dural arteriovenous fistulas (dAVFs) are rare vascular lesions. In this video, the authors describe the case of a 71-year-old female who presented with profound myelopathy secondary to a sacral spinal dAVF. Identification of these lesions requires a high index of clinical suspicion, and a complete spinal angiography study including evaluation of the pelvic vasculature is necessary to detect the arteriovenous shunting. The video demonstrates a nuanced approach to angiographic detection of the dAVF and identification of the fistulous point at the left S1–2 neural foramen, and subsequent treatment with microsurgical selective venous disconnection.

The video can be found here: https://stream.cadmore.media/r10.3171/2025.7.FOCVID25112

## Transcript

### 0:27 Clinical History.

This video presents the microsurgical treatment of a symptomatic sacral spinal dural arteriovenous fistula (dAVF). Patient is a 71-year-old female without any significant past medical history who was transferred from an outside institution. She had presented to the outside hospital with a 48-hour history of worsening bilateral lower extremity weakness and numbness. A Foley catheter was placed at the outside hospital, and this showed urinary retention. She had been experiencing waxing and waning lower extremity weakness and sensory changes for several months.

### 0:53 Neurological Examination.

On neurological examination, she had profound weakness in bilateral lower extremities and reduced sensation to light touch and pinprick below the umbilicus. She had reduced sensation in her perineal area and absent rectal tone.

### 1:08 Imaging.

MRI of the thoracic and lumbar spine revealed T2 hyperintensity extending from the level of T8 to the conus medullaris. There were also prominent flow voids dorsal to the lower thoracic cord and cauda equina on T2-weighted images with enhancement on T1 postcontrast sequences suspicious for abnormal vasculature.

### 1:28

The constellation of clinical symptoms and imaging findings was suspicious for a spinal dAVF, and a spinal angiogram was recommended.

### 1:39

Spinal angiogram demonstrated a type 1 spinal dAVF. Arterial supply was from a radicular branch of the lateral sacral artery arising from the right internal iliac artery and crossing over across midline with the fistulous connection along the nerve root sleeve at the left S1–2 neural foramen. The arterialized draining vein courses superiorly along the spinal canal with perimedullary venous engorgement along the lower thoracic spinal cord and conus medullaris.

### 2:11 Treatment.

After discussion of treatment options, patient elected for microsurgical treatment given higher probability of long-term occlusion.

She was positioned prone, with cranial end toward the right side. Neuromonitoring with SSEPs, MEPs, and sphincter EMGs was performed. Standard exposure of the laminae was performed. Levels were confirmed with fluoroscopy. Bone cuts were made with an ultrasonic scalpel and laminae of L5, S1, and S2 were removed. As expected, lower lumbar and sacral dura was paper thin. Dura was opened sharply in standard fashion and tacked up.

### 2:45

An arterialized vein was seen extending superiorly along the left side of the spinal canal. The overlying nerve roots were sharply dissected, and the arterialized vein was followed inferiorly and found to arise from the dura at the left S1–2 neural foramen, as expected based on the angiogram. An ICG videoangiography was performed which showed early venous drainage, thereby confirming arterialized nature of the vein.

### 3:20

A mini aneurysm clip was applied to the vein close to the exit site at the foramen. We waited about 20 minutes to ensure there was no change in neuromonitoring data. The vein was then coagulated. Fluorescein videoangiography showed resolution of early venous drainage. Due to the large sacral spinal canal, we elected to leave the mini aneurysm clip in situ.

### 3:56

The dura was approximated with running 6-0 Gore-Tex sutures followed by a layer of DuraSeal Exact. After hemostasis, S1–2 laminae, which had been removed en bloc, were fixed back in place with laminoplasty plates and screws. A lumbar drain was inserted through the dura at the level of the L5 laminectomy. The incision was then closed in a layered fashion.

A postoperative spinal angiogram was obtained, which did not demonstrate any residual arteriovenous shunting on selective lateral sacral artery injection or pelvic flush angiography, thereby indicating cure of the fistula.

### 4:25 Clinical Outcome—Immediate.

Patient began to improve neurologically while in the hospital, with increased motor strength and improvement in sensation, and was discharged to inpatient rehabilitation on postoperative day 8.

### 4:38 Clinical Outcome—4 Months Postop.

By delayed follow-up at 4 months postoperatively, she had return of full strength in her lower extremities and was able to climb up and down stairs. She had residual decreased sensation in both lower extremities. Her bladder function had improved with only occasional urinary incontinence. Her bowel movements had returned to normal.

### 4:56 Discussion.

Spinal dAVFs are rare vascular lesions.^1,2^ For patients presenting with myelopathic symptoms and no clearly identifiable etiology, it is important to have a high index of suspicion for dAVFs or other vascular shunting lesions.^1^ When a standard thoracolumbar spinal digital subtraction angiography (DSA) does not demonstrate a lesion, additional imaging extending to the cervical or sacral vasculature should be performed to ensure a comprehensive evaluation. Noninvasive imaging can often help guide spinal DSA.^3^

### 5:30

Treatment options for spinal dAVFs include endovascular embolization with liquid embolic agents or microsurgical ligation. Endovascular embolization has the advantage of being minimally invasive but has lesser long-term occlusion rates, as penetration of the embolic agent into the venous compartment of the fistula, which is critical for cure, can often be challenging.^2,4^ Microsurgical treatment is more invasive but provides definitive treatment of the fistula with selective venous disconnection.^5,6^ Patient-related factors and dAVF anatomy should be considered when determining the optimal treatment strategy.

With timely diagnosis and intervention, patients with severe neurological deficits can attain significant neurological improvement, as seen in this case.

## Disclosures

The authors report no conflict of interest concerning the materials or methods used in this study or the findings specified in this publication.

## Author Contributions

Primary surgeon: Vellimana. Editing and drafting the video and abstract: both authors. Critically revising the work: both authors. Reviewed submitted version of the work: Vellimana. Approved the final version of the work on behalf of both authors: Vellimana. Supervision: Vellimana.

## Supplemental Information

### Patient Informed Consent

The necessary patient informed consent was obtained in this study.

## References

[1] Klopper HB Surdell DL Thorell WE Type I spinal dural arteriovenous fistulas: historical review and illustrative case Neurosurg Focus 2009 26 1 E3 10.3171/FOC.2009.26.1.E3 19119889

[2] Patsalides A Santillan A Knopman J Tsiouris AJ Riina HA Gobin YP Endovascular management of spinal dural arteriovenous fistulas J Neurointerv Surg 2011 3 1 80 84 21990796 10.1136/jnis.2010.003178

[3] Saraf-Lavi E Aristizabal J Yavagal DR The role of MRA as a preliminary diagnostic tool in diagnosing and localizing spinal dural arteriovenous fistula (SDAVF) AJNR Am J Neuroradiol Published online May 9, 2025 10.3174/ajnr.A8830 PMC12629581 40345857

[4] Voldřich R Charvát F Malík J Netuka D Evaluating the role of Onyx embolization in the management of spinal dural arteriovenous fistulas: a 20-year single-center experience World Neurosurg 2025 198 124016 40294791 10.1016/j.wneu.2025.124016

[5] Musmar B Roy JM Orscelik A Comparative efficacy and safety of endovascular versus surgical treatment in spinal dural arteriovenous fistulas: a systematic review and meta-analysis Spine (Phila Pa 1976) 2025 50 8-562 10.1097/BRS.0000000000005226 39835409

[6] Garcia-Garcia S Baric H Niemela M Surgical management of spinal dural arteriovenous fistulae: systematic review of current practices and open questions Acta Neurochir (Wien) 2024 166 1 464 39560755 10.1007/s00701-024-06360-z

